# A Systematic Scoping Review of Media Campaigns to Develop a Typology to Evaluate Their Collective Impact on Promoting Healthy Hydration Behaviors and Reducing Sugary Beverage Health Risks

**DOI:** 10.3390/ijerph18031040

**Published:** 2021-01-25

**Authors:** Vivica I. Kraak, Katherine Consavage Stanley

**Affiliations:** Department of Human Nutrition, Foods, and Exercise, Virginia Polytechnic Institute and State University (Virginia Tech), Blacksburg, VA 24061, USA; kconsavage@vt.edu

**Keywords:** typology, media campaign, social change, sugary beverages, water, healthy hydration

## Abstract

Interventions to discourage sugary beverages and encourage water consumption have produced modest and unsustainable behavioral changes to reduce obesity and noncommunicable disease risks. This systematic scoping review examined media campaigns to develop a typology to support healthy hydration nonalcoholic beverage behaviors. Our three-step methodology included the following: (1) review and summarize expert-recommended healthy beverage guidelines; (2) review six English-language electronic databases guided by PRISMA to describe existing campaign types by issue, goal and underlying theory; and (3) develop a media campaign typology to support policies, systems and environments to encourage healthy hydration behaviors. Results showed no international consensus for healthy beverage guidelines, though we describe expert-recommended healthy beverage guidelines for the United States. Of 909 records identified, we included 24 articles describing distinct media campaigns and nine sources that defined models, schemes or taxonomies. The final media campaign typology included: (1) corporate advertising, marketing or entertainment; (2) corporate social responsibility, public relations/cause marketing; (3) social marketing; (4) public information, awareness, education/ health promotion; (5) media advocacy/countermarketing; and (6) political or public policy. This proof-of-concept media campaign typology can be used to evaluate their collective impact and support for a social change movement to reduce sugary beverage health risks and to encourage healthy hydration behaviors.

## 1. Introduction

A robust evidence base shows that adequate and healthy hydration behaviors are essential to promote optimal human health outcomes [1]. The frequent and excessive consumption of sugary beverage products by individuals and populations is associated with adverse diet and health outcomes, including dental caries, obesity and type 2 diabetes [2,3,4,5]. Sugary beverages include different types and brands of soft drinks, sports and energy drinks, fruit drinks and sweetened teas, coffees and milks [3].

Temporal trends in sugary beverage intake by children and adolescents in 15 countries (1990 to 2020) documented that consumption was highest before 2000, and only slight reductions were observed for these populations over 30 years [6]. Evidence is inconsistent on whether the long-term consumption of sugary and artificially sweetened beverages is linked to a higher cardiovascular disease mortality risk in adults [7,8]. Prospective cohort studies suggest that replacing a daily serving of a sugary beverage with water, coffee or tea, but not an artificially sweetened beverage, is associated with a 2 percent to 10 percent lower type 2 diabetes risk [9].

Governments in countries worldwide have enacted policies and programs to define sugary beverage categories and products to limit or discourage their purchase and use. Examples of policies include enacting sugary beverage taxes, requiring manufacturers to place warning labels on sugary beverages to increase awareness about their contribution to obesity and diet-related health risks and establishing nutrition standards for beverage sales in institutions such as schools, while also encouraging healthier beverages to reduce obesity and noncommunicable disease (NCD) risks [10,11,12,13,14,15,16]. However, well-funded corporate campaigns have opposed these government and advocacy public policy efforts and used the media to influence the views of decisionmakers and the public in many countries to challenge the need for these types of policies [12,14,15]. Additionally, many interventions designed to reduce sugary beverage or increase water purchase and intake have had limited to modest effects to sustain behavioral changes to significantly reduce population health risks [17,18].

### 1.1. Trends in the Corporate Marketing of Beverage Brands and Products

Media campaigns have delivered corporate advertising slogans to encourage sugary beverage purchases and intake by individuals since the late 1880–1890s by The Coca-Cola Company [19] and PepsiCo, Inc. [20], two companies that currently dominate beverage markets and sales in over 200 countries worldwide [21]. In 2019, Coca Cola ranked first and PepsiCo ranked sixth as the most chosen brands by consumers worldwide [22].

The Coca-Cola Company and PepsiCo, Inc. together market beverage brands across five categories: sparkling soft drinks or sugary beverages (e.g., Coke, Sprite, Pepsi, Gatorade and Mountain Dew); water (e.g., Dasani, Smartwater, Aquafina, Bubbly and LIFEWTR); juices (e.g., Minute Maid, Fanta, Sunkist and Tropicana); dairy and plant-based drinks (e.g., Fairlife, Simply Almond and Muscle Milk); and coffees or teas (e.g., Costa, Lipton, Pure Leaf and Starbucks ready drinks) [23,24]. Nestlé is the third largest beverage manufacturer that markets many brands in 187 countries including: ready-to-drink chocolate, tea and coffee (e.g., Milo, Nesquik, Ovaltine and Nestea); water (e.g., Nestlé Pure Life, Perrier, Poland Spring and Pellegrino); and specialized toddler milks [25].

The global beverage market is projected to generate $1.86 trillion by 2024 [26]. Transnational beverage firms have partnered with chain restaurants in primarily industry self-regulated markets where national governments have allowed sugary beverages to be widely marketed and socially normalized. These firms have built and sustained consumer demand through advertising delivered across media to encourage sugary beverage consumption, which is linked to obesity and diet-related NCDs in populations worldwide [2,3,4,5].

### 1.2. Mass Media Campaigns to Reduce Obesity and Noncommunicable Disease (NCD) Risks

Mass media campaigns have been used by public health practitioners and policymakers in countries worldwide to promote healthy dietary and physical activity messages to prevent obesity and NCDs among children, teens and adults. Evaluations have shown that these media campaigns have focused primarily on changing an individual’s awareness, knowledge or attitudes about obesity as a problem rather than influencing the upstream environmental factors that perpetuate obesogenic environments [27]. Studies reveal that media campaign effectiveness depends on the issue, message content and audience targeted [27,28]. Many public awareness campaigns have not changed parental attitudes about the causes and consequences of childhood obesity, or have not effectively mobilized public support for broader policies, systems and environmental strategies to address obesity [29]. Campaign planners must determine the specific behaviors to target and how to frame communications persuasively to increase the perceived message effectiveness among target audiences to adopt and sustain behaviors to improve health outcomes [27].

Stead et al. 2019 conducted an exhaustive review of media campaigns to change behaviors to reduce obesity and NCD risks [30] and concluded that evidence was limited for the effectiveness of diet-related media campaigns, while results were mixed for media campaigns to effectively reduce sedentary behaviors and promote physical activity. These investigators suggested that future evaluations should examine how campaigns contribute to multicomponent interventions and explore how local, regional and national campaigns work synergistically to achieve a clear goal [30].

Media campaigns have been used to influence beverage behaviors [14,17,18] but many have not been evaluated. A 2019 Cochrane systematic review of environmental interventions to reduce sugary beverage sales and consumption included only multicomponent media campaigns with a control community as part of the evaluation design [31]. An in-depth evaluation of the design, outputs, outcomes and impact of beverage-related media campaigns has not yet been published.

Reviews of mass media campaigns to promote public health outcomes suggest that they are more likely to be effective when planners apply recommended principles, test and develop persuasive messages for targeted populations, adequately fund and execute campaigns as planned to disseminate behaviorally focused messages, update messages as evidence is revised, set realistic milestones and expectations for the media campaign outcomes and expect small to modest behavioral effects for targeted populations [30,32,33,34]. Best-practice recommendations encourage campaign planners to clarify the situation and priorities linked to a vision and mission [35] and to convey actionable, credible, relevant, trusted and understandable messages [36]. Campaign planners must also understand the target audiences’ awareness about the health risks of behaviors, their knowledge about policies to mitigate the risks and their confidence to take recommended actions [36].

### 1.3. Mass Media Campaigns to Support Social Change Movements

Social change is a long-term process that fosters collective action to transform social norms, attitudes and behaviors of populations for a specific issue over years or decades [37,38]. Figueroa et al. 2002 [38] identified mass media campaigns as one of many factors used to catalyze community dialogue to influence individual and collective actions within a social change process. Social change movements have harnessed print, broadcast and digital media and used social networking platforms to deliver persuasive messages through media campaigns to influence social norms, attitudes, beliefs, values, behaviors and actions to benefit populations [37,38].

Advocacy campaigns implemented in the United States (U.S.) during the 19th and 20th centuries evolved into effective social movements by harnessing the collective social and political actions of people to improve urban housing conditions in cities; demand higher wages and reduce working hours; enhance children’s health and social welfare; and discourage substance use [39]. Social change movements have often been led by people most affected by health inequities or racial or gender biases to challenge existing narratives, assumptions and paradigms about power and privilege in societies and disrupt the status quo [37,38].

Examples of effective media campaigns used to support social change movements include those that enacted tobacco marketing policies to restrict availability in public settings; promoted environmental conservation and recycling behaviors; required transportation safety with seat belt laws for automobiles and helmet use for bicycles and motorcycles; and protected civil and gender rights and racial and health equity for historically disenfranchised populations [37,38,39,40,41].

The tobacco prevention and control movement in the U.S. that expanded globally is one of the most effective social change movements that used mass media campaigns with a broader policy, systems and environmental (PSE) approach to transform cultural values and behaviors to reduce tobacco use and smoking rates from the 1950s to 2010 [42,43]. The U.S. Food and Drug Administration’s Real Cost public information campaign [44], American Legacy Foundation’s Truth social marketing campaigns [45], the Tobacco-Free Generation proposal [46] and the Tobacco Endgame strategy [47] have used media to build on previous public policy and social change achievements to prevent the sale and supply of tobacco products and socially de-normalize and discourage tobacco and vaping products among young people. The concept of creating a Sugary Beverage-Free Generation has been described [48], but it has not been fully explored to understand how media campaigns can be used as part of a broader PSE approach to discourage sugary beverage products and encourage and socially normalize drinking water and other healthy hydration behaviors among populations.

### 1.4. Study Purpose

The purpose of this review is to develop a coherent media campaign typology to examine how different types of campaigns can support broader PSE approaches to reduce sugary beverage health risks and encourage healthy hydration behaviors. There is limited published literature on how different types of media campaigns can support a social change movement to discourage sugary beverages and normalize healthy beverage intake as part of a broader PSE approach that includes sugary beverage taxes and warning labels to benefit population health. We also sought to examine expert-recommended healthy beverage guidelines for children, adolescents and adults to have a reference point for analyzing the campaign messages when the typology is tested in the U.S. context.

This study addresses these issues with three objectives. First, to summarize expert-recommended guidelines issued by the World Health Organization (WHO) and other international and U.S. expert and authoritative bodies to encourage healthy hydration nonalcoholic beverage behaviors for populations. Second, to conduct a systematic scoping review of relevant evidence to identify types of media campaigns used to influence awareness, preferences, purchase and consumption behaviors of alcohol, tobacco, food and/or beverage products; and to identify conceptual models, taxonomies, typologies and categorization schemes used to delineate or categorize media campaigns. Third, to use these campaign definitions, models, taxonomies and typologies to develop a unique media campaign typology that can be used to describe the goals and underlying paradigms for each campaign type. Thereafter, we plan to test this typology in the U.S. context to examine the evidence to evaluate the collective impact of beverage-related media campaigns to encourage healthy beverage behaviors. The typology may support a Sugary Beverage-Free Generation social movement, similar to the Tobacco-Free Generation proposal, to encourage healthy hydration behaviors to reduce sugary beverage health risks.

## 2. Materials and Methods

This study used a systematic scoping review process guided by three research questions (RQ).

RQ1: What are existing international and U.S. recommended guidelines or targets to encourage healthy hydration beverage behaviors and targets for individuals and populations?
RQ2: What types of conceptual models, taxonomies, typologies or categorization schemes have been used to identify and categorize media campaigns aimed to influence awareness, preferences, purchase and/or consumption behaviors for alcohol, tobacco, food and/or beverage products?
RQ3: How can existing models, taxonomies, typologies or categorization schemes be adapted into a media campaign typology to evaluate the collective impact of media campaigns on policies, systems and environments to support a social change movement to establish a Sugary Beverage-Free Generation?

### Search Strategy, Evidence Selection, and Evidence Extraction for RQ1 and RQ2

RQ1 provides relevant background information for media campaigns to promote healthy beverage behaviors. To address RQ1, the principal investigator (VIK) used the Google search engine (up to 100 search hits) to conduct a rapid scoping review of healthy beverage recommendations issued by authoritative or expert bodies between 1 January 2000 and 1 December 2020. Websites were searched for grey-literature reports published by and accessed through the World Health Organization (WHO), the six WHO Regional Offices, the Food and Agriculture Organization (FAO) of the UN that describes countries that have translated their national dietary guidelines into food-based dietary guidelines, and the World Cancer Research Fund’s Continuous Update Project and NOURISHING database.

VIK also searched U.S. government agencies responsible for developing healthy beverage guidelines, including Health and Human Services (HHS), U.S. Department of Agriculture (USDA) and the Centers for Disease Control and Prevention (CDC); private foundations, including the Robert Wood Johnson Foundation’s Healthy Eating Research National Program; nongovernmental organizations (i.e., National Academy of Medicine and American Heart Association); and professional health societies and organizations (i.e., American Academy of Pediatrics, American Dental Association, Academy of Nutrition and Dietetics, American Society for Nutrition, American Public Health Association and the American Medical Association). The results were independently reviewed by the co-investigator (KCS) to identify expert-recommended guidelines and targets for healthy beverages that could be used in media campaigns to prevent dental caries and reduce obesity and NCD risks.

To address RQ2, the co-investigators (VIK and KCS) identified six published reviews of media campaigns used to discourage sugary beverages or encourage heathy beverages to identify search terms and develop a “working media campaign typology” that included: (1) corporate or commercial advertising and marketing, corporate social responsibility or cause marketing campaigns; (2) social marketing campaigns; (3) public information, awareness, health education or health promotion campaigns; (4) counteradvertising or media advocacy campaigns; and (5) political or public policy campaigns. We also reviewed persuasion theories, models and frameworks used to evaluate persuasive communications [49] that served as a foundation for the campaign designs.

KCS worked with university research librarians to design a search strategy for a systematic scoping review of evidence that described the purpose, characteristics and underlying paradigms and theories of media campaigns that have been used to influence the awareness, preferences, purchase and/or consumption behaviors of alcohol, tobacco, food and beverage products. We selected a systematic scoping review because of the broad nature of the research questions and to more comprehensively examine the evidence gap for this topic. Many expert recommendations for sugar beverages are unlikely to be initially published in peer-reviewed literature, and we were aware of multiple stakeholder groups that produce relevant reports and other grey literature for campaigns. Likewise, evidence quality was not an initial priority as we were solely focused on identifying distinct categorizations, models, typology or taxonomies and we anticipated a qualitative evidence synthesis [50].

We followed five steps for the scoping review described by Arksey and O’Malley 2005 [51] to examine the peer-reviewed literature and grey-literature sources. We identified articles that described a conceptual model, typology, taxonomy or categorization scheme either within one type of media campaign or across different types of media campaigns. The search strategy used the Preferred Reporting Items for Systematic Review and Meta-Analysis Protocol (PRISMA-P) [52] and the PRISMA Extension for Scoping Reviews (PRISMA-ScR) checklist [53]. We did not plan to assess study quality or risk of bias given the exploratory and conceptual nature of the research questions.

The search period established was the database inception to 1 September 2020. We reviewed six English-language electronic databases (i.e., PubMed, Web of Science, PsycInfo, Political Science Complete, Academic Search Complete and Health Source Complete: Consumer and Nursing/Academic editions); and conducted a separate review of Google Scholar (first 100 search hits) for evidence relevant to RQ2. Supplemental Table S1 summarizes the pre-defined search terms used for the title and abstract searches.

RQ2 inclusion criteria were: (1) English-language peer-reviewed review articles, book chapters or grey-literature reports; (2) evidence sources that proposed a unique conceptual model, taxonomy, typology or categorization scheme to understand or categorize a campaign that aimed to influence awareness, preference, purchase and/or consumption behaviors for alcohol, tobacco, foods and/or beverages; and (3) evidence sources that described a model, category, framework, taxonomy or typology for health media campaigns more broadly and use examples of food, beverage, alcohol and tobacco campaigns. RQ2 exclusion criteria were: (1) non-English-language evidence sources; (2) evidence sources that presented primary research findings or evaluations for specific media campaigns; and (3) evidence sources that did not describe or analyze health-related media campaigns for alcohol, tobacco, food or beverages (i.e., safety or HIV/AIDS prevention) or did not discuss a distinct conceptual model, taxonomy, typology or categorization scheme to inform the development of the media campaign typology.

Step one involved KCS reviewing the full-text articles and creating an evidence table to summarize the first author’s last name and year published; the campaign issue(s) examined; type(s) of media campaigns described; any typology or scheme and the basis for its development; target audiences of the media campaign type; underlying theory or theories of change; and documentation of the effectiveness or impact of the media campaigns. VIK also conducted backward searches on inclusion articles to identify any additional relevant evidence sources and separate hand searches using our proposed campaign typology themes to identify any other potentially relevant sources. Step two involved the two co-investigators independently and then collectively analyzing the results to refine our working media campaign typology based on the evidence sources analyzed. The co-investigators discussed and resolved any disagreements with interpreting the article or report for inclusion or exclusion. We address RQ3 in the Discussion section by synthesizing insights from RQ1 and RQ2 to refine our working media campaign. We discuss how it can be used to evaluate the collective impact of media campaigns on policies, systems and environments to support a social change movement to establish a Sugary Beverage-Free Generation.

## 3. Results

This section describes the evidence synthesized for the three research questions as a narrative review. The first section describes evidence for international and U.S. expert-recommended beverage guidelines and targets to encourage healthy beverage behaviors. The second section presents evidence used to develop and refine the categories of our working media campaign typology. The third section synthesizes the evidence to describe how existing models, typologies, taxonomies or categorization schemes can be adapted and operationalized into a media campaign typology to evaluate the collective impact of media campaigns on policies, systems and environments to support a social change movement to establish a Sugary Beverage-Free Generation.

### 3.1. Scoping Review of Recommended Guidelines to Encourage Healthy Beverage Behaviors

#### 3.1.1. International Healthy Hydration Beverage Guidelines or Recommendations, 2000 to 2020 

The WHO recommends that children and adults should limit their consumption of foods and drinks that contain high amounts of free sugars, including all types of sugary beverages, throughout the life course to less than 5–10 percent of total energy intake [54]. The WHO also recommends that national governments implement the WHO’s 2010 recommendations to restrict the marketing of unhealthy food and nonalcoholic beverage products to children and adolescents through age 18 years [55] that includes targets for beverages. Between 2015 and 2019, six WHO Regional Offices released nutrient-profile models for national governments to develop policies and laws to restrict the marketing of unhealthy food and nonalcoholic sugary beverage products to children [56,57].

Between 2000 and 2019, the WHO had updated quality standards for governments to ensure safe drinking water for populations [58]. However, we were unable to identify any WHO Regional Office reports that provided technical guidelines to Member States for healthy hydration beverage categories, targets or behaviors that could be used in media campaign messages and applied across countries, regions or globally as part of a comprehensive obesity and NCD prevention and management plan. The FAO has identified 100 countries that have translated their national dietary guidelines into food-based dietary guidelines, and a recent review documented varied recommendations for nonalcoholic beverages (i.e., sugary, fruit juices, milk and nondairy substitutes) across countries [59]. In 2018, the World Cancer Research Fund International’s evidence update, published by scientific researchers, recommended that people limit their consumption of sugary beverages and drink mostly water or unsweetened beverages but found insufficient evidence to recommend artificially sweetened beverages containing non-nutritive sweeteners [60].

#### 3.1.2. U.S. Healthy Beverage Guidelines, 2000 to 2020

The Dietary Guidelines for Americans (DGA) 2000, 2005 and 2010 [61,62,63] recommended that people moderate their sugar and calorie intake from beverages to maintain a healthy body weight but did not specify daily beverage targets for water, milk or 100 percent juice. In 2013, USDA issued the Smart Snacks in School Standards that recommended school administrators provide children with portion sizes based on their age and established beverage targets, including plain water (carbonated or uncarbonated); unflavored low-fat milk and milk alternatives; 100 percent fruit and vegetable juices; and full-strength juices diluted with water (carbonated or noncarbonated) and with no added sweeteners [64].

The U.S. National Academy of Medicine (formerly called the Institute of Medicine) recommended quantitative Dietary Reference Intake targets in 2005 for total water, beverage water (20 percent from food intake), milk and 100 percent juice for healthy individuals and populations that varied by age and sex across the lifespan [65]. These targets were considered when the DGA developed the sugary beverage recommendations. In 2006, an Expert Beverage Panel comprised of U.S. academic researchers published a seminal paper that proposed a new beverage guidance system that offered quantitative daily targets for various beverage categories that prioritized beverages with few or no calories, including water, tea, coffee, low-fat or non-fat milk and non-calorically-sweetened beverages that may provide some nutritional benefits, to replace sugary beverages [66].

In 2013 and 2019, the U.S. Healthy Eating Research National Program, supported by the Robert Wood Johnson Foundation, released principles and recommended beverage targets for water, milk and 100 percent juice (but not artificially sweetened beverages) throughout the life course (Table 1) [67,68]. The guidelines for infants, toddlers and children (birth to five years old) were updated in 2019 and endorsed by four U.S. health organizations and professional societies. These are the most comprehensive targets for different beverage categories for infants, toddlers and preschoolers, older children, youth and adolescents and adults. These recommendations have not been widely disseminated, and it is not known whether U.S. media campaign messages have incorporated these recommendations.

The DGA 2015–2020 recommended that individuals aged two years and older consume water, fat-free or low-fat milk and/or 100% juice [69]; and USDA’s ChooseMyPlate—the pictorial version of the DGA—showed milk as the default beverage with meals (though not explicitly low-fat or non-fat milk) rather than water [70]. The DGA 2015–2020 also recommended that Americans consume ≤10 percent of their daily calories from added sugars (i.e., 200 calories for a 2000-calorie diet) [69], which was translated by the American Heart Association (AHA) into a recommendation of ≤6 teaspoons or 25 g of added sugars daily for children 2–18 years and adult women, and ≤9 teaspoons or 37.5 g of added sugars daily for adult men [71].

The DGA 2015–2020 acknowledged that high-intensity sweeteners (including artificial sweeteners approved by the Food and Drug Administration, such as aspartame and stevia) could be used but noted that there is insufficient evidence to support their long-term use for weight management [69]. An AHA expert advisory committee encouraged Americans to consume primarily water (plain, carbonated or unsweetened); and issued a qualified recommendation that children with diabetes could substitute low-calorie sweetened beverages for sugary beverages and adults could replace sugary beverages with low-calorie sweetened beverages to reduce overall sugar intake [72].

The 2020 Dietary Guidelines Advisory Committee report [73] recommended that Americans reduce their added sugars to ≤ six percent of daily energy intake. However, the DGA 2020–2025 report did not incorporate the advisory committee’s recommendation and upheld the <10 percent of calories from added sugars included in the DGA 2015–2020 report. The new DGAs also recommended that parents establish a healthy beverage pattern for infants and toddlers under age two years and that adults consume water or other calorie-free choices as their primary beverage—or other nutrient-dense choices, including fat-free and low-fat milk and 100 percent juice [74]. The DGA 2020–2025 lacked specific guidelines for healthy beverages for adults and suggested that individuals “meet their food groups needs with nutrient-dense foods and beverages; limit foods and beverages higher in added sugars, saturated fat and sodium; and limit alcoholic beverages” [74]. No recommendation was made regarding the use of low- or no-calorie high intensity sweeteners in beverages due to questions about their long-term effectiveness as a weight management strategy [74]. 

### 3.2. Evidence Selected for the Systematic Scoping Review to Address RQ2

**Figure 1:**
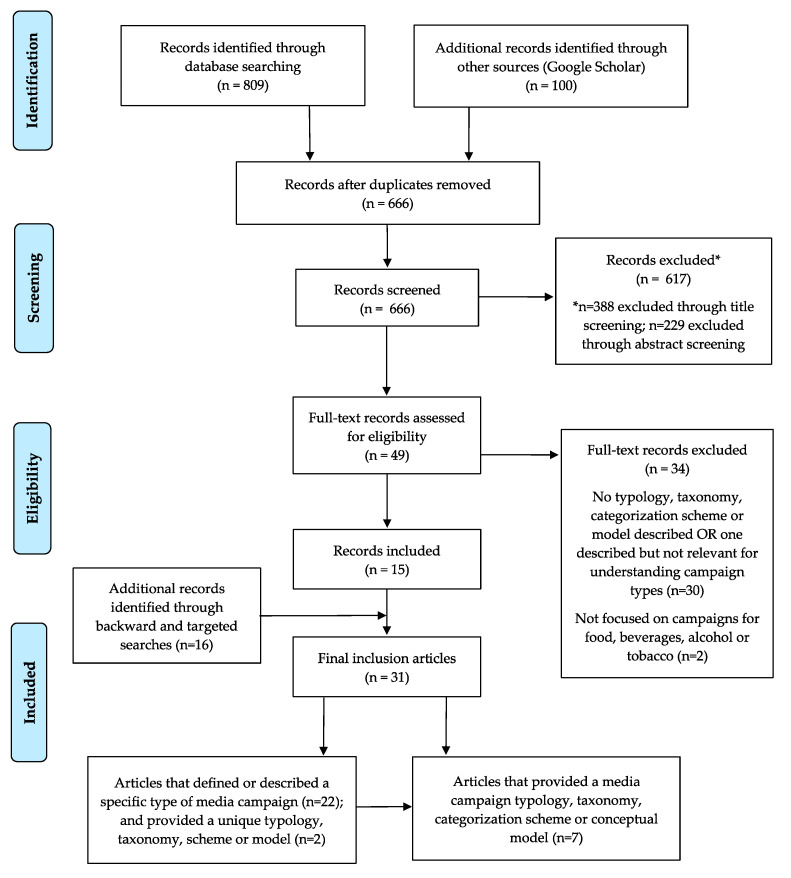
PRISMA flow diagram for the systematic scoping review of evidence for media campaign models, taxonomies, typologies and categorization schemes.

Figure 1 shows the PRISMA flow diagram for the systematic scoping review used to address RQ2. We identified a total of 909 records: 809 records from the six electronic databases and 100 records identified through the Google Scholar search engine. After removal of duplicate records, we screened 666 records, of which 49 full-text records were assessed for inclusion between August and September 2020. We excluded 34 records and selected 15 articles that met the inclusion criteria. An additional 16 articles were identified through a supplemental Google search and by hand searching the reference lists of the included articles between October and November 2020. We included 31 evidence sources in the final review, including 24 articles that defined or described a specific type of media campaign [75,76,77,78,79,80,81,82,83,84,85,86,87,88,89,90,91,92,93,94,95,96,97,98]. Two of these articles [80,95] defined one or more types of campaigns that also qualified as a unique categorization scheme. Seven additional articles, grey-literature reports and media websites described a distinct media campaign model, categorization scheme, typology or taxonomy [79,99,100,101,102,103,104,105].

### 3.3. Media Campaign Descriptions: Goals and Aims or Objectives

The systematic scoping review identified 31 evidence sources published between 1990 and 2020 described below and summarized in Table 2 (*n* = 24 articles) and Table 3 (*n* = 9 evidence sources), with two articles providing relevant information for inclusion in both tables. We organized the campaigns into the five categories of our “working” media campaign typology that we refined after our analysis. We also noted underlying theories for the media campaigns when reported.

#### 3.3.1. Corporate or Commercial Advertising and Marketing, Corporate Social Responsibility or Cause Marketing Campaigns

Four articles described various types of media campaigns used by tobacco, alcohol, food and/or sugary beverage companies and industry trade associations to promote brands and products to populations to maximize revenues and commercial profits [80,82,96,98]. These included using traditional and social media for advertising and marketing campaigns, corporate social responsibility campaigns, cause marketing campaigns and public relations campaigns.

Cruz et al. 2019 [80] examined pro-tobacco advertising and marketing campaigns that used media to tailor package design and messages to reach and appeal to various socio-demographic groups. These investigators defined tobacco marketing as a broad term including paid advertising and promotions in movies and television; sponsorship or loyalty programs; and product design or pricing used by tobacco manufacturers and distributors to reach racially and ethnically diverse populations [80].

Dorfman et al. 2012 [82] described corporate social responsibility (CSR) campaigns and social media cause-marketing campaigns, noting that the latter are a variation of CSR campaigns, whereby a company associates its corporate brand and/or product(s) to a social benefit, such as supporting the development of sports centers at schools. CSR campaigns of soda companies were launched in response to increased public concerns about the health risks of sugary beverage products, and also to increase beverage sales among young people. In contrast, tobacco CSR campaigns that were launched to defend their corporate reputation but not to increase the sales of tobacco products [82].

Weishaar et al. 2016 [96] analyzed how the corporate campaigns of tobacco, alcohol, soft drink and processed food companies are portrayed by the media. These investigators found that commercial actors frame media messages as individual responsibility for obesity and NCDs, and unhealthy consumption or lifestyle behaviors as a personal choice. Moreover, industry actors also portray government regulation as producing negative economic implications based on a “market justice” frame; whereas public health advocates frame messages with regard to how these corporate actors’ production and marketing practices harm human health and serve as systematic causes of NCDs and poor health outcomes. Public health actors use a social justice frame to advocate for government regulation and population-based interventions to improve health outcomes. None of the articles reported underlying theories to explain the design or influence of these campaigns. Wood et al. 2019 [98] examined two Coca-Cola Company advertising campaigns to highlight how beverage companies use public relations to strategically communicate with customers and use brand marketing to target children, adolescents and their mothers through social media platforms. None of the articles reported theories to explain the design or influence of these campaigns.

#### 3.3.2. Social Marketing Campaigns

Nine articles provided definitions for social marketing programs or campaigns used by public health and nongovernmental organizations [75,78,79,81,84,85,88,93,94]. Overall, social marketing involves adapting commercial marketing principles, strategies and techniques to analyze, plan and evaluate programs or campaigns to influence the voluntary behaviors of target audiences. Stead et al. 2007 [93] defined social marketing as adapting commercial marketing principles, strategies and techniques to analyze, plan and evaluate programs or campaigns to influence the voluntary behaviors of target audiences to improve their personal welfare and that of society. Luca and Suggs 2013 [88] described features that distinguish a social marketing campaign from other campaigns and interventions that use the commercial marketing-mix framework to identify a product, price, place and promotion.

Several articles described social marketing campaigns used to raise awareness and encourage the adoption of healthy eating behaviors, such as increasing fruit and vegetable intake. Evans et al. 2008 [84] and Evans et al. 2015 [85] described branded health campaigns more broadly and noted that branding can be used in both health communication and social marketing programs. The definition provided for health branding that emphasized using marketing principles to encourage behavior change most closely aligned with social marketing campaigns.

Shawky et al. 2019 [92] examined whether social marketing campaigns used social media, defined as tools and platforms for social interaction including digital and web-based and mobile technologies, to augment the messages of social marketing campaigns. These investigators found that Facebook was the primary social media platform used by only a small proportion of campaigns to share content to reach target audiences in order to raise awareness, influence health behaviors and encourage advocacy for campaign activities.

Only three articles described specific theories or frameworks for the social marketing campaigns. Cugelman et al. 2011 [81] and Luca and Suggs 2013 [88] described social marketing as drawing on many theories, including the transtheoretical theory or stages of change model, social cognitive theory, cognitive behavioral therapy, health belief model, diffusion theory and the theory of reasoned action. Stead et al. 2007 [93] indicated that a social marketing framework is based on many disciplines and theories to explain human behavior and described six steps to benchmark a social marketing campaign summarized in Table 2. These authors noted that using the term “social marketing” is inconsistent among health interventions and found many interventions that used it but did not meet the six benchmark criteria for social marketing campaigns, while other interventions were not called social marketing but met the criteria [93].

#### 3.3.3. Public Information, Awareness, Health Education or Health Promotion Campaigns

Six articles described media campaigns used to share public information to educate and raise awareness among target individuals and populations about the hazards of using alcohol and tobacco products [80], raise awareness and educate about reducing sugary beverages [94] and dietary salt [95] or encourage individuals and populations to engage in healthy lifestyle behaviors [77,95,97]. Bouman and Brown 2010 [77] described that lifestyle campaigns could promote health by building awareness and influencing the public’s attitudes, beliefs, values or behaviors. Three articles noted at least one kind of theory or framework used in the design of health education [95]; public information [97]; and lifestyle campaigns [77].

#### 3.3.4. Counteradvertising or Media Advocacy Campaigns

Five articles described either countermarketing campaigns used by public health advocacy groups to counteract the commercial advertising and marketing messages of tobacco, alcohol and sugary beverages corporations and reduce demand for products [76,89,90]; or media advocacy campaigns used to challenge or influence corporate industry practices that harm health [86] and to target policymakers and citizens to drive policy change to address the social determinants of health [83].

Bellew et al. 2017 [76] described how social countermarketing is a social change process that starts at the community level and is focused on public good. These authors noted that while social countermarketing campaigns are often created to oppose certain policy positions or commercial marketing tactics that may be harmful to the health or well-being of people and society as a whole, they can also be used to counteract potentially harmful sociocultural norms that have been created through persistent corporate influence [76]. Dorfman et al. 2014 [83] emphasized that with media advocacy campaigns, the focus is not on delivering public health messages to the general public to change behaviors for better health outcomes but is instead more narrowly focused on using media to elevate dialogue and mobilize individuals to put pressure on policymakers, with the ultimate goal of eliciting policy change that will improve the health and well-being of the wider population Only three articles reported on theory: one article reported on agenda setting and media framing theories [83], one on an integrative social countermarketing framework to explain the influence of these campaigns [76] and one mentioned how social cognitive theory helped to understand the influence of peer engagement in tobacco campaigns on individuals’ behavior [90].

#### 3.3.5. Political or Public Policy Campaigns

Only one article, Iyengar and Simon 2000 [87], emphasized the importance of using conceptual models for political campaigns in order to consider the relationship between message content and the predispositions of the targeted populations as well as the interactions between competing campaign messages. These authors noted that political and public policy campaigns may be used to either support or undermine social change and described three theoretical models that can be used to identify and understand the objectives of political campaigns: the resonance model, the strategic model and the traditional model (summarized in Table 2) [87].

### 3.4. Description of Media Campaign Models, Schemes, Taxonomies and Typologies

Table 3 summarizes nine distinct models, categorization schemes, typologies or taxonomies across one or more types of media campaigns. Two of the sources were included in both Table 2 and Table 3. Three typologies were relevant to corporate actors that described the purpose, motives or views about corporate branding [101]; used different types of corporate social media marketing campaigns to reach customers, improve a company’s reputation and maximize sales [103]; and used corporate misinformation or disinformation campaigns to influence decisionmakers or deceive the public about scientific research that produced consequences for public health and safety [104].

Five models, schemes or typologies described various public information or health promotion media campaigns that could be implemented by nonprofit organizations and health organizations: a public relations communication campaign typology [99]; a model that described pro-tobacco marketing media campaigns in order to develop more effective anti-tobacco education campaigns [80]; a multicomponent campaign characterization scheme that combined public awareness and health education to influence the behaviors of targeted populations [95]; a campaign typology that influenced target audiences through three different behavioral changes [102]; and a taxonomy for a public communication campaigns defined by purpose, scope and maturity [100]. One typology described a multicomponent media advocacy and public policy campaign to encourage government regulation of industry marketing practices targeted to children [106].

Schroeder 2017 [101] described a corporate branding typology with four branding motives to understand how businesses “articulate, embody and embrace cultural contradictions and corporate strategy.” The typology includes a (1) corporate view to develop a firm’s brand strategy to build awareness, recognition, engagement and loyalty; (2) consumer view focused on the role of brands in customers’ lives and in consumer culture exemplified by brand relationships and brand tribes; (3) cultural view where brands are embedded in culture and have meaning, history and a legacy; and a (4) critical view that reveals how brands function as ethical, ideological and political objects [102].

ThriveHive 2017 [103] also offered a typology for corporate campaigns, describing four kinds of social media marketing campaigns that individuals or corporations can use to reach large groups of people with a specific product or message. The typology consists of (1) partnership campaigns, (2) holiday campaigns, (3) milestone campaigns and (4) charity campaigns (described in more detail in Table 3) [103].

Union of Concerned Scientists 2018 [104] provided a categorization scheme for understanding five corporate campaign tactics that some companies use to undermine or distort science and public health and to protect their own interests. These tactics—given the nicknames the fake, the blitz, the diversion, the screen and the fix—have been used by corporations to cause scientific confusion and ambiguity and blur the lines between science, policy and corporate interests [104].

Bünzli and Eppler 2019 [99] defined public relations as a strategic communications process used by nonprofit organizations to build trust and mutually beneficial and sustainable relationships with the public to achieve goals. Public relations may take many forms, including organizational communication, employee communications, media relations, community relations, corporate and social responsibility, public affairs, crisis management and social media. The authors describe four categories of public relations matched to different stages of change (based on the trans-theoretical model) to systematize different types of communication approaches that organizations use when interacting with the public to encourage target audiences to adopt a specific behavior: directing, platforming, involving and mobilizing (see Table 3 for category descriptions) [79].

Snyder et al. 2004 [102] conducted a meta-analysis of the effect of U.S. health communication campaigns categories based on their desired effect on behavior to: (1) prevent an undesirable behavior, (2) start a new behavior or (3) stop an existing behavior. Using the diffusion theory, these authors maintain that it is easier to convince people to begin a new behavior rather than to prevent an undesirable behavior or stop a current behavior (especially if it is an addictive behavior), and that campaigns that include enforcement messages are more successful at influencing behavior [102]. As a new behavior becomes socially normalized and more people engage in it, positive role models and public health campaigns may reinforce behavioral changes. However, campaign planners should establish realistic goals because health campaigns have small measurable short-term effects on population behaviors [102].

Watson and Martin 2019 [105] describe a multicomponent media campaign typology that combined media advocacy with encouraging citizen protest and engagement and developing relationships with decisionmakers to build political support for government regulation of industry practices that undermine population health. These investigators describe the important role of civil society organizations to mobilize the public and the media to support health policies, and recommend sustained campaigns given the long-term nature of driving health policy change [105].

## 4. Discussion

This systematic scoping review examined the available evidence to develop a media campaign typology to support healthy hydration nonalcoholic beverage behaviors. We used three steps to identify relevant evidence summarized in Table 1, Table 2 and Table 3. Thereafter, we synthesized the evidence into a unique media campaign typology. Our analysis for RQ1 revealed a lack of universally accepted, expert guidance for healthy hydration behaviors and targets for nonalcoholic beverage categories across the life course. We discussed several recommended guidelines issued by U.S. expert and government bodies, and found that the Healthy Eating Research provides the most comprehensive healthy beverage guidelines for different age groups (Table 1). 

Given the lack of comprehensive guidance or a clear definition for healthy hydration behaviors by U.S. and international bodies, we propose a working definition for healthy hydration behaviors. Healthy hydration behaviors prioritize the purchase and/or consumption of water, low- or non-fat milk and other healthier beverage choices outlined in the Healthy Eating Research recommendations (Table 1). We also propose the promotion of water as the default healthy beverage choice. We recommend that the WHO harmonize national and international guidelines for Member States to normalize healthy beverage behaviors for populations, delivered through food-based dietary guidelines and promoted through media campaigns in geographically diverse countries, regions and globally.

**Figure 2:**
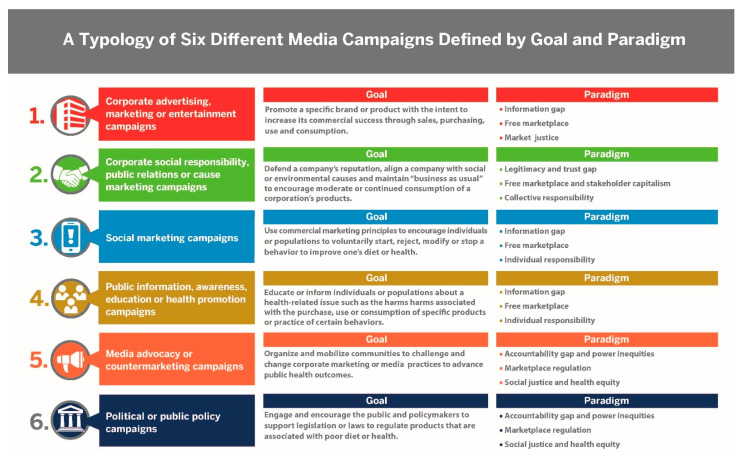
A typology of six different media campaigns defined by the goal and paradigm.

Figure 2 defines the goal and underlying paradigm for each campaign in our proposed typology based on the evidence analysis and synthesis. The final typology included: (1) corporate advertising, marketing or entertainment campaigns; (2) corporate social responsibility, public relations or cause marketing campaigns; (3) social marketing campaigns; (4) public information, awareness, education or health promotion campaigns; (5) media advocacy or countermarketing campaigns; and (6) political or public policy campaigns.

Media—in digital, broadcast and print forms—is a major component of our typology as the key vehicle through which these campaigns share their messages and aim to influence large audiences in order to reach their broader campaign goal. Mass media frames public health policy debates to influence the public’s views by selecting which issues to report and how they are discussed [106]. Mass media also frames the nature and drivers of public health problems and potential policy solutions [106]. Weishaar et al. 2016 [96] and Henderson and Hilton 2018 [106] discuss how corporate actors use a “market justice” media frame versus public health advocacy actors who use a “social justice” media frame to justify their actions in the marketplace. These contrasting frames reflect different values and underlying paradigms associated with media. It is important to analyze how the media deliver campaign messages, as well as analyze the institutions that produce and use the media, and the impact on actor networks, practices, technologies and political ideologies [106].

We provide illustrative examples of U.S. beverage campaigns below that fit into the proposed campaign typology based on different goals and underlying paradigms. These examples will require further analysis based on available evidence for campaign evaluations. The first category for corporate media campaigns combines advertising, marketing and entertainment to promote a specific brand or product with the intent to increase its commercial success through sales, purchasing, use and consumption. U.S. media advertising campaign examples include: The Coca-Cola Company’s Open Happiness (2009–2015), Polar Bears Catch (2012) and Share a Coke and a Song (2015) [19]. Popular PepsiCo, Inc. campaigns include The Choice of a New Generation (1984), Pepsi for Every Generation (2018) and For the Love of It (2019) [20].

The second category includes public information, awareness, education or health promotion campaigns designed to educate or inform individuals or populations about a health-related issue, such as the harms associated with the purchase, use or consumption of specific products or with practicing certain behaviors. Examples of U.S. beverage campaigns include: Rethink Your Drink (2011–2020), Life’s Sweeter Without Sugary Drinks (2011), The Bigger Picture (2013) and The Sour Side of Sweet (2017) [92,96,107,108,109]. Future evaluations should examine whether the framing of health messages in these media campaigns have led to meaningful changes in cognitive or behavioral outcomes for populations. A Cochrane review of health-message framing suggests that the message goal and attributes have had limited or no consistent effect on consumers’ behaviors [110].

The third category includes social marketing campaigns where planners have used commercial marketing principles to encourage individuals and populations to voluntarily begin, reject, modify or stop a behavior to improve their diet or health. U.S. beverage campaign examples include: 1% or Less to encourage low-fat milk (1990s); Got Milk and Milk Mustache? (1995–2015); and Drink Up (2013) to promote water [111]. Given the inconsistent use of “social marketing” within existing campaigns and interventions, we recommend that campaign planners follow the guidelines and benchmark criteria set out by existing social marketing researchers [79,93].

The fourth category includes corporate cause marketing, CSR and public relations campaigns. Dorfman et al. 2012 [82] and Wood et al. 2019 [98] describe the purpose of these campaigns as associating a company’s brands or products with a social benefit and communicating with target populations to defend a corporate reputation in response to concerns about their products. Schroeder 2017 [101] and ThriveHive 2019 [103] describe many ways that corporations use branding and social media marketing campaigns to achieve their goals. This category is different from the corporate advertising, marketing and entertainment campaigns because CSR, cause marketing and public relations campaigns are used to defend a company’s reputation, align a company with a cause to positively influence the public’s perceptions of the company and/or maintain “business as usual” to encourage moderate or continued consumption of branded products or introduce new products.

Examples of U.S. cause marketing, CSR and public relations media campaigns are the Coca-Cola Company’s Live Positively (2010), Movement is Happiness (2013), The Great Meal and Together Tastes Better (2020) and Together We Must (2020) racial justice dialogue campaigns to support the #BlackLivesMatter movement [82,100,112,113]. PepsiCo, Inc. has also used CSR and public relations campaigns such as the Pepsi Refresh Project (2010) that provided grants to local communities using social media [83]. In 2020, PepsiCo, Inc. launched a combined CSR, cause marketing and public relations campaign that linked social justice and environmental recycling commitments through Black Art Rising, which sponsored visual Black artists connected with a new branded water product called LIFEWTR packaged in 100% recyclable plastic containers and promoted through a hashtag social media campaign [114]. 

PepsiCo, Inc. also launched the Food for Good “purpose-driven” initiative to address food insecurity and hunger among U.S. children in response to the coronavirus (COVID-19) in 2020 by partnering with local communities to distribute PepsiCo products [115]. Both Coca-Cola Company and PepsiCo have also used CSR and public relations in partnership with the American Beverage Association to promote the Mixify campaign (2014) that was part of the U.S. Balance Calories Initiative (2014) designed to reduce the calories consumed by Americans from sugary beverage products nationwide by 20 percent by 2025 [116].

The fifth category includes media advocacy or countermarketing campaigns that share the goal to organize and mobilize communities to challenge corporate marketing practices to advance public health outcomes. U.S. beverage campaign examples include: Kick the Can (2012), The Real Bears (2012), Happiness Stand and Coming Together: Translated (2013), Open Truth (2015), Share a Coke with Obesity and Change the Tune (2015) campaigns [90,94,117].

The sixth category includes political or public policy campaigns that use media to engage the public and policymakers to support legislation or laws to regulate products associated with poor diet and health. Watson and Martin 2019 [105] identified the need for a multicomponent campaign to support political and social change that uses media advocacy to frame an issue and potential solutions, encourage citizen protest and engagement and develop relationships with policymakers and decisionmakers to build political support for government regulation. Kennedy et al. 2018 use the term “upstream social marketing” to influence structural and systems change through policymakers [118]. Examples of U.S. beverage campaigns that fit into this category are ones that successfully enacted legislation and local ordinances to support sugary beverage taxes in seven U.S. cities or municipalities, including Berkeley and Oakland, California and Boulder, Colorado [10,12,119].

**Figure 3:**
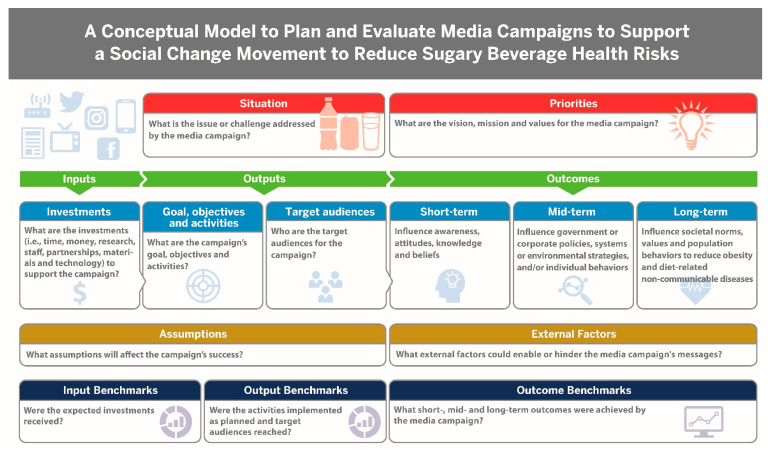
A conceptual model to plan and evaluate media campaigns to support a social change movement to reduce sugary beverage health risks. *Adapted from:* Foster, B.; Horton, B.; DeFrancesco, L.; Wedeles, J. *Evaluating Social Change.* Washington, DC: Vanguard Communications Purple Paper; 2012 (reference [35]).

Foster et al. 2012 [35] suggest that planners use a theory of change framework or conceptual model to identify the long-term goal and objectives of media campaigns and the pathways needed to achieve them and recommends clarifying the evaluation processes before implementation. Planners should also identify the assumptions and external factors that may undermine campaign effectiveness; and monitor and evaluate indicators of success for inputs, outputs and outcomes [35]. Figure 3 provides a conceptual model to plan and evaluate media campaigns to support a social change movement to reduce sugary beverage health risks. Our proposed typology is an important tool for planners because a media campaign strategy must examine the competing messages from corporate advertising and marketing campaigns that may undermine public health campaigns prior to implementing and when evaluating a media campaign.

Future research should examine the content and perceived effectiveness of messages reported by media campaigns, and the alignment of the campaign messages with government- and expert-recommended beverage targets that promote healthy eating patterns. No published study has examined how media campaigns have been used to support policies, systems and environmental strategies to discourage sugary beverage sales and consumption, while promoting water and low-fat milk choices and other healthy beverage behaviors among populations.

To address RQ3, we discuss how this typology may be used to evaluate the collective impact of campaigns on policies, systems and environments to support a social change movement to establish a Sugary Beverage-Free Generation. A collective impact approach involves developing a common agenda and shared measurement as well as providing mutually reinforcing activities, continuous communication and organizational support for the entire initiative [120]. Effective collective impact must consider “who is engaged, how they work together and how progress happens” [121]. A collective impact evaluation approach is better positioned to address large-scale complex societal challenges, such as sugary beverage overconsumption that contributes to dental caries, obesity and diet-related NCDs. We believe that this proof-of-concept media campaign typology may support the strategic communications for a Sugary Beverage Free Generation, which could draw from the Tobacco-Free Generation social change movement that has built support across several Asian countries to phase out the sale and use of tobacco products among young people [122,123,124].

### 4.1. Cross-Cutting Issues for All Media Campaigns

Several topics emerged during the evidence screening and analysis that represent cross-cutting issues relevant to the design, implementation and evaluation of different media campaigns to achieve public health and social change outcomes. These issues include: (1) planning for unintended effects or consequences of media campaigns, (2) harnessing social media used by all campaign types and (3) anticipating that media campaigns may spread misinformation or disinformation and produce both intended and unintended effects for audiences.

Dillard et al. 2018 suggest that using fear or scare tactics to influence beverage-related health behaviors may lead audiences to resist or reject media campaign messages that may require counter-persuasion to encourage healthy behaviors [125]. Cho and Salmon 2007 developed a typology of 11 unintended side effects of public health communication campaigns including: obfuscation (creating confusion or misunderstanding of a health risk or prevention behavior), dissonance (psychological distress based on the difference between health recommendations and an individual’s experience), boomerang (audience reaction is opposite to the intended response), apprehension (unnecessary concern about health in response to the health-risk message), desensitization (public may not respond after repeated exposure to the health messages), culpability (messages are perceived as blaming individuals rather than the social or environmental circumstances), opportunity cost (selection of certain health issues over others to improve the population’s health), social reproduction (campaigns that reinforce existing knowledge and behaviors among different audiences), social norming (marginalization of certain populations disproportionately impacted by health risks), enabling (promote images and finances of industries or improve the power of individuals and institutions), and system activation (the campaign may influence unintended sectors, and may mediate or moderate the campaign effects on the intended audiences) [126].

Social media is used across different campaign types [83,88,94,104], which is a major social innovation that has changed how humans communicate, with important implications for public relations, advertising, marketing and business [127] as well as public health advocacy and policies [41,84,106,128]. Recent political campaigns have used micro-targeting through social media platforms that are tailored to the demographic and psychographic profiles of voters to influence them to support or reject certain political candidates or policy issues [129]. Personalization and microtargeting strategies are used with virtual social media hashtag campaigns such as Share a Coke and a Song, whereby the company offered customers personalized bottles of Coca Cola to increase sales while building an emotional relationship and brand loyalty [130]. Social media platforms can be used to launch either small- or large-scale campaigns to motivate people to take steps toward health-related behavior changes [131]. Social media indicators should be measured as part of the overall campaign evaluation [129].

Various institutional actors may use social media campaigns to spread misinformation and disinformation. One typology that we identified described five corporate strategies used to promote misinformation (the unintentional sharing of information) or disinformation (the intentional spreading of misleading political, reputational or financial information) to prioritize business interests over public health and safety [104]. Krishna and Thompson 2019 [132] and the WHO [133] state that misinformation campaigns spread false or scientifically inaccurate information and impede people’s health literacy to make appropriate decisions. These types of campaigns have been used extensively by tobacco firms [134]. Future research is needed to examine how beverage companies use these types of campaigns to influence the public and policymakers about the link between beverage products and diet-related health outcomes, and how these campaigns may influence their views about corporate legitimacy and accountability for marketing practices.

### 4.2. Study Strengths and Limitations

This is the first study to develop a unique typology to understand how different media campaigns are used to influence awareness, preference, purchase and/or consumption behaviors for alcohol, tobacco, foods and/or beverages. The typology describes each media campaign based on its unique goal and underlying paradigms. A strength is the use of both peer-reviewed articles and grey-literature sources to develop the typology that allowed us to identify corporate strategies and media campaign tactics not readily available in the published literature. Another strength is the compilation of existing healthy hydration guidance from U.S. and international expert bodies that may inform our analysis of media campaign messages used in the U.S. context to promote healthy hydration behaviors. However, future research should examine whether international consensus exists for healthy beverages since we did not conduct a comprehensive review of all existing international guidelines for this topic. There is also a need to differentiate between corporate media campaigns used to promote sugary beverage brands and products linked to obesity and NCD risks from branded functional beverages emerging in the marketplace that may provide phytonutrients that support healthy dietary patterns [135]. Study limitations are the use of solely English-language articles and the inability to include evidence sources that did not clearly define or describe interventions used in various types of campaigns. An additional limitation was the difficulty to accurately describe distinct media campaigns given the evolving media and marketing landscape as the boundaries between the various campaign types have become blurred.

## 5. Conclusions

Interventions to discourage sugary beverages and encourage water consumption have produced modest and unsustainable behavioral changes to reduce obesity and NCD risks. This systematic scoping review examined evidence for expert-recommended beverage guidelines. The WHO should harmonize national and international guidelines that clearly define sugary beverage products and help countries to establish national guidelines and targets for healthy beverage behaviors for various populations that can be promoted through media campaigns in geographically diverse countries, regions and globally. We also reviewed the evidence for existing campaigns to develop a unique media campaign typology that has six distinct categories: (1) corporate advertising, marketing or entertainment campaigns; (2) corporate social responsibility, public relations or cause marketing campaigns; (3) social marketing campaigns; (4) public information, awareness, education or health promotion campaigns; (5) media advocacy or countermarketing campaigns; and (6) political or public policy campaigns. This proof-of-concept campaign typology can be used to evaluate the collective impact of media campaigns on policies, systems and environments in a country, region or globally for a specific social, environmental, diet or health issue. This typology should be tested to evaluate whether it may promote healthy hydration behaviors and support a social change movement to establish a Sugary Beverage-Free Generation.

## Figures and Tables

**Table 1:** U.S. Healthy Eating Research healthy beverage recommendations by beverage category and age group.

Beverage Category	Infants and Toddlers 0–5 Years	Children 6–10 Years	Tweens and Teens 11–18 Years	Adults > 19 Years
Plain Water	0–6 months: none 6–12 months: 0.5 to 1 cups/day 12 months–3 years: 1 to 4 cups/day 4–5 years: 1.5 to 5 cups/day * *No added sweeteners or carbonation*	Includes plain and carbonated water with no added sweeteners and access to free, safe drinking water wherever beverages are served or sold	Includes plain and carbonated water with no added sweeteners and access to free, safe drinking water wherever beverages are served or sold	Includes plain and carbonated water with no added sweeteners and access to free, safe drinking water wherever beverages are served or sold
Milk	0–12 months: none 12–24 months: 2 to 3 cups/day whole milk 2–3 years: ≤ 2 cups/day skim or low-fat milk 4–5 years: ≤ 2.5 cups/day skim or low-fat milk	Includes unflavored, low-fat and non-fat milk with no added sweeteners and soy beverages (calcium and vitamin D fortified) in no more than 8-ounce (oz) portions	Includes unflavored, low-fat and non-fat milk with no added sweeteners and soy beverages (calcium and vitamin D fortified) in no more than 12-ounce portions	Includes unflavored, low-fat and non-fat milk with no added sweeteners and soy beverages (calcium and vitamin D fortified) in no more than 12-ounce portions
100% Juice	0–12 months: none 1–3 years: ≤4 ounces/day 4–5 years: 4 to 6 oz/day	0 to 6 oz/day portions of 100% fruit or vegetable juice or fruit juice combined with water, no added sweeteners, and no more than 100 mg sodium per portion	0 to 8 oz/day portions of 100% fruit or vegetable juice or fruit juice combined with water, no added sweeteners, and no more than 140 mg sodium per portion	0 to 8 oz/day portions of 100% fruit or vegetable juice or fruit juice combined with water, no added sweeteners, and no more than 140 mg sodium per portion
*Guiding Principles*	*All beverages should be free of synthetic food dyes, stimulants (i.e., caffeine) and other additives (e.g., electrolytes and artificial flavors).*			
Other Beverages	Not recommended: flavored milk, plant milks only for medical or dietary purposes, toddler milk, sugary beverages, beverages with low-calorie sweeteners and caffeinated beverages.		Noncaffeinated, nonfortified beverages with no more than 40 calories per container.	Low- to mid-calorie beverages with no more than 40 calories per container. Pre-packaged coffee or tea beverages with no more than 40 calories per container. If coffee or tea are prepared onsite, the milk should be low-fat or nonfat with no added caloric sweeteners in no more than 12-oz portions.

*Sources:* Healthy Eating Research. *Recommendations for Healthier Beverages.* March 2013. https://healthyeatingresearch.org/wp-content/uploads/2013/12/HER-Healthier-Bev-Rec-FINAL-3-25-13.pdf; and Healthy Eating Research. *Healthy Beverage Consumption in Early Childhood. Key Recommendations from Key National Health and Nutrition Organizations.* September 2019. https://healthyeatingresearch.org/wp-content/uploads/2019/09/HER-HealthyBeverage-ConsensusStatement.pdf (references [67,68]).

**Table 2:** Evidence from the scoping review of specific types of media campaigns for alcohol, tobacco, food and/or beverages defined by goal, aim or objective and underlying theory or conceptual framework, 1990–2020.

First Author, Year Published	Goal and Issue	Type of Media Campaigns Purpose, Aims or Objectives	Theory or Conceptual Framework
**1. Corporate or commercial advertising and marketing, corporate social responsibility, cause marketing or public relations campaigns (*n* = 4)**				
Cruz et al. 2019 [80]	Promote or reduce tobacco product use among vulnerable populations.	**Pro-tobacco marketing campaigns:** Used by tobacco companies to increase tobacco sales and use among individuals and populations.	Not reported
Dorfman et al. 2012 [82]	Promote or reduce soda and tobacco product use and consumption.	**Corporate social responsibility (CSR) campaigns:** Use print, broadcast, digital and social media to promote or align a company with social causes or benefits to address economic, legal, philanthropic or ethical issues. CSR campaigns aim to improve the company’s reputation among stakeholders, including legislators, consumers, government regulators and the media. **Cause marketing campaigns:** Purpose-driven or brand-purpose initiatives that link a brand or product to a social benefit, often in partnerships with companies or a nonprofit organization to provide a portion of revenue for a social, health or environmental cause.	Not reported
Weishaar et al. 2016 [96]	Describe how marketing and media campaigns are used by tobacco, alcohol, soft drink and processed food companies.	**Corporate advertising and marketing campaigns:** Used by beverage firms and industry trade associations to promote beverage brands to targeted populations; encourage “choices” among their product portfolios; and use a “market justice” frame to rationalize corporate practices that promote sugary beverages to populations.	Not reported
Wood et al. 2019 [98]	Describe how food and beverage companies use media in corporate public relations campaigns.	**Corporate public relations campaigns:** Soda companies use public relations to develop relationships with customers, including mothers and children, build allies and oppose or marginalize the opposition.	Not reported
**2. Social marketing campaigns (*n* = 9)**				
Aschemann-Witzel et al. 2012 [75]	Design more effective public health campaigns to promote healthy foods and dietary behaviors.	**Public health campaigns that use social marketing:** Use of commercial food marketing success factors to promote voluntary behavior change among target audiences to improve healthy eating behaviors.	Not reported
Brambila-Macias et al. 2011 [78]	Promote healthy eating behaviors to increase fruit and vegetable intake and reduce dietary salt or sodium intake.	**Public information/social marketing campaigns:** Use commercial marketing practices to influence voluntary behavior change among individuals and populations.	Not reported
Cavicchi et al. 2011 [79]	Promote healthy foods to influence dietary behaviors of individuals and populations.	**Social marketing campaigns:** Adapt commercial marketing principles for social outcomes and uses behavioral theories, targeting and community- or individual-level involvement.	Not reported
Cugelman et al. 2011 [81]	Design online interventions that deliver messages to improve population adherence to change health behaviors.	**Social marketing health behavior change campaigns:** Use techniques and principles that often involve incentives to encourage a target audience to voluntarily accept, modify, reject or stop a specific behavior in order to benefit an individual, group or society.	Transtheoretical theory; social cognitive theory; cognitive behavioral therapy; behavioral therapy; extended parallel process model; health belief model; and the theory of reasoned action.
Evans et al. 2008 [84]	Promote healthy behaviors through media viewed by children and parents.	**Social marketing campaigns:** Use commercial marketing principles to promote health behavior change at an individual or population level.	Not reported
Evans et al. 2015 [85]	Reduce tobacco use and consumption and promote a healthy diet by increasing fruit and vegetable intake.	**Branded public health campaigns:** Use social marketing principles to promote and support behavior change and create symbols or identities that embody pro-social and health-promoting behaviors.	Marketing, psychological and communication theories.
Luca and Suggs 2013 [88]	Stop tobacco use and smoking behaviors and make dietary changes to reduce heart disease risks.	**Social marketing campaigns:** Used to influence a target audience to either start, reject, modify or stop a specific behavior in order to benefit the individual, specific groups or society and produce social change.	Economic exchange theory; diffusion theory; theory of planned behavior; theory of reasoned action; health belief model; protection and motivation theory; stages of change or Transtheoretical model; social cognitive theory
Shawky et al. 2019 [92]	Use social media in campaigns to support participant engagement in social marketing programs.	**Social marketing and social media campaigns:** Use social media (e.g., tools and platforms for social interaction such as digital, web-based and mobile technologies) to augment traditional media to raise awareness about issues and make communications accessible, interactive and scalable to raise target audience awareness about health issues.	Not reported
Stead et al. 2007 [93]	Influence individual behavior, environmental and policy change for tobacco, drugs, alcohol and physical activity.	**Social marketing campaigns:** Applies commercial marketing principles, strategies and techniques to analyze, plan and evaluate programs or campaigns to influence the voluntary behaviors of target audiences to improve their personal welfare and societal outcomes.	Social marketing framework is based on many disciplines and theories to explain human behaviors. Social marketing framework has six components to benchmark a campaign or program: (1) behavior change; (2) consumer research; (3) segmenting and targeting populations; (4) marketing mix (product, place, price and promotion); (5) exchange; and (6) competition.
**3. Public awareness, information, education or health promotion campaigns (*n* = 6)**				
Bouman and Brown 2010 [77]	Raise awareness and influence behaviors to encourage healthy lifestyles.	**Lifestyle campaigns:** Use media and/or entertainment education to communicate messages to targeted audiences over a period of time to raise awareness about a health issue, and/or influence attitudes, beliefs, values or behaviors as part of a lifestyle.	Communitarian ethical framework
Cruz et al. 2019 [80]	Reduce tobacco product use among vulnerable populations.	**Anti-tobacco public education campaigns:** Use media to promote educational messages to reduce tobacco use.	Not reported
Randolph et al. 2012 [91]	Use campaigns to discourage and stop tobacco use among individuals and populations.	**Health promotion campaigns:** Initiatives that aim to increase uptake of healthy behaviors by changing people’s knowledge about a health behavior, attitudes towards a health behavior and/or adoption of the behavior.	Not reported
Te et al. 2019 [94]	Use social media to disseminate health messages about sugar-sweetened beverages.	**Social media anti-sugary beverage health campaigns:** Use educational materials and activities created by a health institution and disseminated through various social media platforms to persuade a target population to reduce their consumption of sugary beverage products to improve diet and health outcomes.	Not reported
Trieu et al. 2017 [95]	Reduce population intake of dietary salt or sodium to decrease cardiovascular disease risks.	**Salt-reduction public awareness and health education campaigns:** Used to deliver information to raise awareness and educate target groups about dietary salt to lower cardiovascular disease risks and improve health outcomes.	Social ecological model; social cognitive theory; self-management principles; principles of behavior change; PRECEDE-PROCEED framework.
Weiss et al. 1994 [97]	Use public information campaigns to support policies.	**Public information campaigns:** Government-directed and sponsored efforts to communicate to the public a achieve a policy outcome.	Policy theory
**4. Counteradvertising or media advocacy campaigns (*n* = 5)**				
Bellew et al. 2017 [76]	Use social countermarketing to create social, environmental and health benefits for people and society.	**Social countermarketing campaigns:** Uses concepts and techniques that contrast commercial (for profit) and social (public good-focused) countermarketing to reach decisionmakers to support social change.	Integrative social countermarketing framework based on theories and models that have an upstream/systems focus combined with theories and models that have a downstream or individual focus.
Dorfman et al. 2014 [83]	Use media advocacy to support public health goals.	**Media advocacy campaigns:** Grounded in social justice values to target policymakers and mobilize individuals to drive policy change and address the social determinants of health.	Agenda-setting and media-framing theories.
Freudenberg et al. 2009 [86]	Change industry practices that damage human health.	**Media advocacy:** Used by one or more organizations to launch targeted activities of varying duration to change specific corporate or industry practices that harm health.	Not reported
McKenna et al. 2000 [89]	Reduce tobacco use among youth.	**Tobacco countermarketing campaigns:** Used to counteract pro-tobacco marketing messages and influence through sharing pro-health messages to influence the behaviors of target audiences.	Not reported
Palmedo et al. 2017 [90]	Reduce tobacco, alcohol and unhealthy food and sugary beverage demand and use among individuals and populations.	**Countermarketing campaigns:** Use health communications to reduce consumer demand for unhealthy products by exposing the motives and undermining the marketing practices of producers leading to changes in industry marketing practices.	Social cognitive theory
**5. Political or public policy campaigns (*n* = 1)**				
Iyengar and Simon 2000 [87]	Use public relations and media advocacy strategies to promote healthy and democratic societies.	**Political campaigns:** Strengthen the relationship between message content and the predispositions of the targeted populations as well as the interactions between competing campaign messages to achieve a policy outcome.	Describes three theoretical models: (1) *Resonance Model* that aligns campaign messages with individuals’ existing preferences. (2) *Strategic Model* that focus on interactions between competing messages. (3) *Traditional Model* where campaign characteristics determine the effect.

**Table 3:** Evidence from the scoping review of unique media campaign models, schemes, typologies or taxonomies, 1990–2020.

First Author, Year Published	Goal and Issue	Description of the Media Campaign Models, Schemes, Typologies or Taxonomies (*n* = 9)
Bünzli and Eppler 2019 [99]	Public communications campaigns used by nonprofit organizations to create social change.	**Public relations and public communications campaign model with four categories based on the communication purpose and communication style:** *Directing:* Educate the audience by sharing their own point of view, not in receiving audience feedback or input. *Platforming:* Educate the audience using active listening to hear audience concerns and ideas and acts as a platform for exchange *Involving:* aim Empower audiences by encouraging dialogue on issues *Mobilizing:* Empower audiences by suggesting specific actions
Cruz et al. 2019 [80]	Examine how media campaigns are used to target populations to promote or reduce tobacco availability and use.	**A model that describes two types of media campaigns to promote or discourage tobacco products.** *Pro-tobacco marketing campaigns:* Use media to market to individuals and populations to increase tobacco sales and use. *Anti-tobacco public education campaigns:* Use media to educate individuals and populations to discourage and reduce tobacco purchases and use.
Dorfman et al. 2002 [100]	Reduce alcohol and tobacco availability and use.	**Public communication campaign typology to reduce alcohol and tobacco use based on three criteria:** *Purpose:* what the campaign is trying to accomplish; purpose is a continuum with personal behavior at one end and policy change at the other. *Scope:* the size and extent of the campaign; continuum with small, targeted on one side and large, broad on the other; and *Maturity:* of the campaign and issue based on a continuum with younger (informal) versus older (formal) campaigns.
Schroeder 2017 [101]	Use of branding by corporations for various purposes including media campaigns.	**Corporate branding campaign typology with four dimensions:** *Corporate view:* Focuses on developing a firm’s brand strategy to build brand awareness, recognition and loyalty; *Consumer view*: Focused on the role of brands and branding in the lives of customers and in consumer culture exemplified by brand relationships, brand community and brand tribes; *Cultural view:* Focused on brands embedded in the culture that have meaning, heritage, history and a legacy rather than being used exclusively as a management tool; and *Critical view* that reveals ways that brands function as ethical, ideological, and political objects.
Snyder et al. 2004 [102]	Reduce alcohol and tobacco use and promote a heathy diet to prevent cardiovascular disease.	**Behavior change campaign typology to reduce alcohol use through three types of behaviors:** Campaigns that promote a new behavior; Campaigns that discourage an old behavior; and Campaigns that prevent a new undesirable behavior.
ThriveHive 2017 [103]	Use social media marketing to achieve several public relations, CSR, advertising and marketing goals.	**Corporate social media marketing campaign typology:** *Partnership campaigns:* Associate a brand with a more successful or better-known brand, raise awareness about both brands, reach an audience currently not targeted by a company’s products or services, and create strong business to business relationships. *Holiday campaigns:* Design an advertising campaign around many holidays. *Milestone campaigns:* Celebrate a company’s anniversary, opening a new business location, launching a new product or service, customer loyalty targets, or reaching a certain number of social media followers. *Charity campaigns:* Support charitable causes and ask staff to volunteer for the company’s charity of choice and document it using social media, such as through Facebook Live streaming.
Trieu et al. 2017 [95]	Use a multicomponent media campaign to reduce population intake of dietary salt or sodium.	**Multi-component media campaign typology combines two types of campaigns to reduce salt intake:** *Public awareness campaigns:* Change population behavior on a large scale and are characterized by short messages delivered through mass media, print and digital media. *Health education programs/campaigns:* Provide information about salt-reduction delivered directly to groups of people.
Union of ConcernedScientists 2018 [104]	Corporate strategies used to promote business interests and undermine public health or safety.	**A corporate disinformation or misinformation campaign typology with five strategies:** *The fake:* Companies conduct counterfeit science and try to pass it off as legitimate research by ghosting writing articles, selectively publishing positive results or commissioning scientific studies biased toward predetermined results. *The blitz:* Companies or industry trade associations that oppose or attack scientists who speak out with results or views that challenge the industry. *The diversion*: Companies that may create scientific uncertainty about evidence that challenges a product’s adverse effects (e.g., tobacco, dietary salt or sugary beverages) by deceiving the public and undermining government regulatory agencies that have a mission to protect the public’s health. *The screen*: Companies may purchase credibility and legitimacy by developing alliances with academic universities or professional societies, or by sponsoring academic positions, students, or fund research. *The fix:* Influence government officials or the policy process.
Watson and Martin 2019 [105]	Reduce unhealthy food and beverage marketing to children through government engagement and regulation.	**A multicomponent campaign typology with three categories to support political and social change.** Use media advocacy to frame an issue and potential solutions, and boost the discussion of an issue; Encourage citizen protest and engagement; and Engage with decisionmakers to build political support for government regulation.

## Data Availability

Not applicable.

## Supplementary Materials

The following are available online at https://www.mdpi.com/1660-4601/18/3/1040/s1, Supplemental Table S1: Search strategy used for the systematic scoping review to develop a media campaign typology to promote healthy hydration beverage behaviors to reduce obesity and noncommunicable disease risks.
