# Supplementary material for: A Systematic Scoping Review of Media Campaigns to Develop a Typology to Evaluate Their Collective Impact on Promoting Healthy Hydration Behaviors and Reducing Sugary Beverage Health Risks
Source: Int J Environ Res Public Health. 2021 Jan 25;18(3):1040. doi: 10.3390/ijerph18031040 (PMC7908303; doi:10.3390/ijerph18031040)
Supplement: Supplementary file 1 [file ijerph-18-01040-s001.pdf]

Supplementary Material

Table S1. Search strategy used for the scoping review to develop a media campaign typology to promote healthy hydration beverage behaviors to reduce obesity and non-communicable disease risks.

Databases	Search Terms
**Academic Search Complete (*n* = 30 articles identified), Political Science Complete (*n* = 18), PsycInfo (*n* = 12) and Health Source Complete: Consumer (*n* = 6) and Nursing/Academic Editions (*n* = 6)**	((TI campaign) OR (AB campaign)) AND ((TI health) OR (AB health)) AND ((AB review) OR (TI review)) AND ((TI model*) OR (AB model*) OR (TI framework*) OR (AB framework*) OR (TI theor*) OR (AB theor*) OR (TI typolog*) OR (AB typolog*) OR (TI taxonom*) OR (AB taxonom*) OR (TI methodol*) OR (AB methodol*) OR (TI technique*) OR (AB technique*) OR (TI concept*) OR (AB concept*) OR (TI criteria) OR (AB criteria) OR (TI classification*) OR (AB classification*)) AND ((AB food*) OR (TI food*) OR (AB snack*) OR (TI snack*) OR (AB beverage*) OR (TI beverage*) OR (AB SSB) OR (TI SSB) OR (AB "energy drink") OR (TI "energy drink") OR (AB soda*) OR (TI soda*) OR (AB juice*) OR (TI juice*) OR (AB milk) OR (TI milk) OR (AB "carbonated water") OR (TI "carbonated water") OR (AB (water AND (consum* OR drink*))) OR (TI (water AND (consum* OR drink*))) OR (AB drinking) OR (TI drinking) OR (AB alcohol*) OR (TI alcohol*) OR (AB liquor) OR (TI liquor) OR (AB tobacco) OR (TI tobacco) OR (AB nicotine) OR (TI nicotine) OR (AB cigar*) OR (TI cigar*) OR (AB vape) OR (TI vape) OR (AB vaping) OR (TI vaping))
**PubMed (*n* = 354 articles identified)**	(campaign) AND (review) AND (health) AND (model* OR framework* OR theor* OR typolog* OR taxonom* OR methodol* OR technique* OR concept* OR criteria OR classification*) AND (food* OR snack* OR beverage* OR SSB OR "energy drink" OR soda* OR juice* OR milk OR "carbonated water" OR (water AND (consum* OR drink*)) OR drinking OR alcohol* OR liquor OR tobacco OR nicotine OR cigar* OR vape OR vaping)
**Web of Science (*n* = 383 articles identified)**	(((TI=campaign) OR (AB=campaign)) AND ((TI=health) OR (AB=health)) AND ((AB=review) OR (TI=review)) AND ((TI=model*) OR (AB=model*) OR (TI=framework*) OR (AB=framework*) OR (TI=theor*) OR (AB=theor*) OR (TI=typolog*) OR (AB=typolog*) OR (TI=taxonom*) OR (AB=taxonom*) OR (TI=methodol*) OR (AB=methodol*) OR (TI=technique*) OR (AB=technique*) OR (TI=concept*) OR (AB=concept*) OR (TI=criteria) OR (AB=criteria) OR (TI=classification*) OR (AB=classification*)) AND ((AB=food*) OR (TI=food*) OR (AB=snack*) OR (TI=snack*) OR (AB=beverage*) OR (TI=beverage*) OR (AB=SSB) OR (TI=SSB) OR (AB="energy drink") OR (TI="energy drink") OR (AB=soda*) OR (TI=soda*) OR (AB=juice*) OR (TI=juice*) OR (AB=milk) OR (TI=milk) OR (AB="carbonated water") OR (TI="carbonated water") OR (AB=(water AND (consum* OR drink*))) OR (TI=(water AND (consum* OR drink*))) OR (AB=drinking) OR (TI=drinking) OR (AB=alcohol*) OR (TI=alcohol*) OR (AB=liquor) OR (TI=liquor) OR (AB=tobacco) OR (TI=tobacco) OR (AB=nicotine) OR (TI=nicotine) OR (AB=cigar*) OR (TI=cigar*) OR (AB=vape) OR (TI=vape) OR (AB=vaping) OR (TI=vaping)))
**Google Scholar (first 100 hits)**	campaign AND review AND health AND (model* OR framework* OR theor* OR typolog* OR taxonom* OR methodol* OR technique* OR concept* OR criteria OR classification*) AND (food* OR snack* OR beverage* OR SSB OR soda* OR drinking OR tobacco OR alcohol*)

The six English-language electronic databases searched in September 2020 were: Academic Search Complete, Political Science Complete, PubMed, PsychInfo, Health Source Complete (Consumer and Nursing/Academic Editions) and Web of Science. The detailed search terms are listed in the table below for each database.
